# Are depression and poor sleep quality a major problem in Turkish Women receiving chemotherapy for breast cancer?

**DOI:** 10.1590/1806-9282.20231377

**Published:** 2024-04-22

**Authors:** Mahmut Buyuksimsek, Ahmet Gulmez, Okan Pirinci, Mert Tohumcuoglu, Mehmet Mutlu Kidi

**Affiliations:** 1 University of Health Sciences, Adana Health Practice and Research Center, Department of Medical Oncology – Adana, Turkey.; 2 University of Health Sciences, Adana Health Practice and Research Center, Department of Internal Medicine – Adana, Turkey.; 3 Cukurova University, Department of Medical Oncology – Adana, Turkey.

**Keywords:** Breast cancer, Depression, Sleep quality

## Abstract

**OBJECTIVE:**

The aim of this study was to evaluate depression and sleep quality in Turkish women receiving neoadjuvant or adjuvant chemotherapy for breast cancer and investigate their relationship.

**METHODS:**

This cross-sectional, descriptive, and analytical study included 183 patients who received chemotherapy for non-metastatic breast cancer. Data were collected using the Beck Depression Inventory-II, the Pittsburgh Sleep Quality Index, and a disease-related/sociodemographic information form.

**RESULTS:**

The mean age of the participants was 50.2 years, and 50.3% were in menopause. The mean Beck Depression Inventory-II score was 19.64±10.4. Mild depression was detected in 25.7% (n=47) of the women, and moderate or severe depression in 55.2% (n=101). The mean global score of sleep quality was found to be 8.28±2.62, and the majority of the participants (79.7%, n=146) had poor sleep quality. There was a positive correlation (p<0.001, r=0.43) between depression and sleep quality scores. While a negative correlation was found between depression scores and age (p<0.001, r=0.26), the surgical procedure performed did not significantly affect depression scores (p=0.705). Additionally, depression scores were positively correlated with sleep duration (p<0.001, r=0.42) and sleep latency (p=0.01, r=0.48).

**CONCLUSION:**

Very high rates of depression and poor sleep quality were detected among Turkish women receiving neoadjuvant or adjuvant chemotherapy for breast cancer. The entire healthcare team involved in the treatment process should take this relationship into consideration and use the necessary preventive and therapeutic methods.

## INTRODUCTION

Breast cancer is the most common type of cancer affecting women across the world, with one in eight women being diagnosed with this disease during their lifetime^1^. Being diagnosed with breast cancer is a major traumatic experience for women due to its effects on self-image and sexual relationships; therefore, patients with breast cancer experience many psychiatric problems during the treatment process. Issues experienced by survivors include existential concerns, psychological reactions, and physical symptoms that can potentially impair their well-being. The overall prevalence of depression among oncology patients remains unclear, with previous studies reporting a prevalence between 0 and 58%. This wide range may be due to the use of different tools and criteria to define and evaluate depression, as well as differences in the disease stage and treatment modalities of the evaluated patients^2^. The prevalence of depression is high during the first year after breast cancer diagnosis. A review emphasized that although the prevalence of depression varied across studies, the highest risk period for depression was the first year following breast cancer diagnosis^3^. Sleep disorders are reported in up to 50% of cancer patients, and women diagnosed with breast cancer experience sleep disorders at higher rates (67–90%) than other cancer patients^4,5^. Depression and sleep disorders frequently occur together in breast cancer patients, and they both increase the negative impact on patients’ quality of life^6^. It is also known that depression and sleep disorders are associated with decreased adherence to treatment and reduced survival among breast cancer patients^7^. Cultural differences in societies may affect depression symptoms, and the relationship between sleep disorders and social and cultural factors is also well established, which can also explain the different results obtained from studies conducted to evaluate depression and sleep quality in cancer patients^8^. In this study, we aimed to evaluate depression and sleep quality in Turkish women receiving adjuvant or neoadjuvant chemotherapy for breast cancer and to investigate the relationship between these two psychological disorders.

## METHODS

This cross-sectional, descriptive, and analytical study was conducted from April 2022 to May 2023. The study population consisted of women with breast cancer who received chemotherapy at the outpatient chemotherapy unit of the University of Health Sciences Adana City Training and Research Hospital (Yuregir, Adana). The study included patients aged ≥18 years with non-metastatic breast cancer who had undergone or were scheduled to undergo any type of mastectomy, whose mastectomy type was determined, and who received neoadjuvant or adjuvant chemotherapy. None of the patients had a history of depression or other mental disorders before the diagnosis of breast cancer. In addition, patients with a history of using sleeping pills or other sedative drugs were excluded from the study. Data were collected using the Beck Depression Inventory-II (BDI-II), the Pittsburgh Sleep Quality Index (PSQI), and a disease-related/sociodemographic information form. The disease-related/sociodemographic information form was used to collect data on age, menopausal status, a family history of breast or other cancer, marital status, educational level, number of children, side of the breast involved, mastectomy procedure, household income, number of chemotherapy sessions, type of chemotherapy (neoadjuvant or adjuvant), a history of depression and sleep disorders, and medication use. The depression status of the patients was determined with the BDI-II scale, which is frequently used for this purpose. The BDI-II scale consists of 21 statements, each scored between 0 and 3 according to their severity. The total score that can be obtained from the scale is 63, with higher scores being associated with a higher level of depression. Scores between 0 and 13 indicate minimal depression; those between 14 and 19 indicate mild depression; those between 20 and 28 indicate moderate depression; and those between 29 and 63 indicate severe depression^9^. The PSQI, developed by Buysse et al., is a reliable tool for assessing sleep quality and disorders in the past month. The PSQI evaluates seven sleep components, namely, sleep disorders, sleep medication use, sleep latency, subjective sleep quality, sleep duration, habitual sleep efficiency, and daytime dysfunction. Each component is scored from 0 to 3, and a maximum of 21 points can be achieved in total. A total score of 5 or higher is indicative of poor sleep quality^10^. This study followed the recommendations of the ethics principles published in the Declaration of Helsinki, developed by the World Medical Association, and approved by the Clinical Research Ethics Committee of Adana City Training and Research Hospital (date: March 24, 2022, meeting number: 102, decision number: 1859). The participants were informed about the purposes and method of the study, and their written consent was obtained. The patients were informed that they could withdraw from the study at any time without giving any reason. The resulting data were coded and analyzed using the SPSS v. 22.0 software (SPSS, Inc., Chicago, IL, USA). Analytical tests (Pearson correlation analysis and chi-square test) and descriptive statistics (mean±standard deviation and percentage) were used during statistical analyses.

## RESULTS

**Figure 1:**
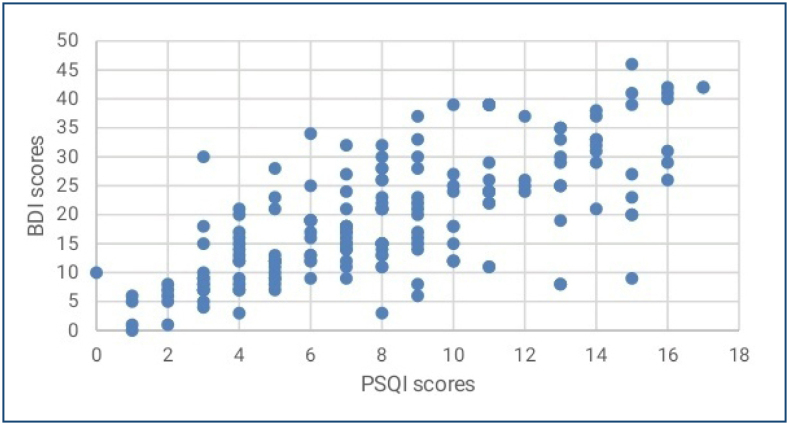
The scatter graph shows a positive, moderate correlation between the Pittsburgh Sleep Quality Index scores and Beck Depression Inventory scores.

A total of 183 patients who met the inclusion criteria were included in the study. The mean age of the patients was 50.2±10.75 (range: 25–75) years. Half of the participants (n=92, or 50.3%) stated that they were in menopause, while 48.1% had a family history of cancer. The sociodemographic and disease-related characteristics of the patients are summarized in Table 1. The mean BDI-II score was 19.64±10.4. Mild depression was detected in 25.7% (n=47) of the women, moderate depression was detected in 36.6% (n=67), and severe depression was detected in 18.6% (n=34). Accepting a global PSQI score of 5 or higher as an indicator of poor sleep quality, the majority of the participants (79.7%, n=146) had poor sleep quality. The mean global score of sleep quality was found to be 8.28±2.62. There was a moderately positive correlation between depression scores and sleep quality scores (p<0.001, r=0.43) (Figure 1). While a negative correlation was found between depression scores and age (p<0.001, r=-0.26), there was a positive correlation between the number of chemotherapy sessions and depression score (p<0.001, r=0.27), and these results were statistically significant. We found a negative correlation between depression scores and the number of children (p=0.887, r=-011) and household income (p=0.31, r=-0.075), but these results did not reach statistical significance. Also, the group receiving neoadjuvant treatment had a statistically significant tendency toward depression compared to the group receiving adjuvant treatment (p=0.03). The depression scores of premenopausal patients were statistically significantly higher than those of menopausal patients (p<0.001). Severe depression was detected in 79.4% of premenopausal patients and in 20.6% of menopausal patients. However, no significant relationship was found between educational level and depression scores (p=0.12). There was also no statistically significant difference in depression scores according to the presence of a family history of cancer (p=0.12) or a family history of breast cancer (p=0.31). Marital status and surgical procedures did not significantly affect depression scores (p=0.143 and 0.705, respectively). Table 2 presents the mean±standard deviation) scores on the overall PSQI and its subscales. There was a significant positive correlation between sleep disturbances and habitual sleep efficiency in breast cancer patients receiving chemotherapy (p<0.001, r=0.33). Additionally, we found a significant positive correlation between the number of chemotherapy sessions and subjective sleep quality (p=0.046, r=0.14). A negative correlation was observed between subjective sleep quality and daytime dysfunction, but this did not reach statistical significance (p=0.6, r=-0.039). Finally, depression scores were positively correlated with sleep duration (p<0.001, r=0.42) and sleep latency (p=0.01, r=0.48).

**Table 1:** Sociodemographic and disease-related characteristics of participants (n=183).

Sociodemographic characteristics			Disease-related characteristics	
**Age**		**(Years, M±SD)**	**Menopause status**	**n (%)**
		50.19	Yes	92 (50.3)
		(±10.75)	No	91 (49.7)
**Number of children**			**Family history of cancer**	n (%)
	0	25 (13.8)		
	1	23 (12.6)		
	2	67 (36.6)	Yes	88 (48.1)
	3	36 (19.7)	No	95 (51.9)
	4	16 (8.7)		
	5	5 (2.7)	**Family history of breast cancer**	n (%)
	6	3 (1.6)	Yes	60 (32.8)
	7	7 (3.8)	No	123 (67.2)
	8	1 (0.5)		
**Marital status**		n (%)		
	Single	8 (4.4)	**Chemotherapy type**	n (%)
	Married	135 (73.8)	Neoadjuvant	82 (44.8)
	Divorced/separated	40 (21.9)	Adjuvant	101 (55.2)
			**Involved breast**	n (%)
**Educational attainment**		n (%)	Right	71 (38.8)
	İlliterate	32 (17.5)	Left	108 (59)
	Elementary school	89 (48.6)	Bilateral	4 (2.2)
	High school	44 (24)		
	University	18 (9.8)	**Type of mastectomy procedure**	n (%)
**Employment**		n (%)	Segmental	87 (47.5)
	Housewife	142 (77.6)	Total	96 (52.5)
	Employee	3 (1.6)		
	Nurse (hemşire)	4 (2.2)	**Number of chemotherapy sessions**	n (%)
	Teacher	12 (6.6)	<5	41 (22.4)
	Secretary	6 (3.3)	>5	142 (77.6)
	Retired	5 (2.7)		
	Textile exporter	3 (1.6)		
	Housekeeper	8 (4.4)		
**Household gross income**		n (%)		
	Good	26 (14.2)		
	Moderate	50 (27.3)		
	Low	107 (58.5)		

M±SD: mean±standard deviation; n (%): number (percent).

**Table 2:** Mean±standard deviation scores on Global Pittsburgh Sleep Quality Index and Pittsburgh Sleep Quality Index Subscales.

Subscales	Mean (±standard deviation)	Range
Subjective sleep quality	1.32 (±0.99)	0–3
Sleep latency	1.17 (±0.96)	0–3
Sleep duration	1.38 (±0.94)	0–3
Habitual sleep efficiency	1.20 (±0.83)	0–3
Sleep disturbances	1.24 (±0.91)	0–3
Use of sleeping medication	1.06 (±0.92)	0–3
Daytime dysfunction	0.93 (±0.92)	0–3
Global PSQI	8.31 (±3.95)	2–21

PSQI: Pittsburgh Sleep Quality Index.

## DISCUSSION

This study aimed to evaluate depression and sleep quality in Turkish women receiving adjuvant or neoadjuvant chemotherapy for breast cancer and investigate their relationship. Approximately 80.9% of the participants reported experiencing mild to severe depression. This rate is significantly higher than the rate (34.7%) reported by Turhal et al., in a healthy Turkish female population^11^. In contrast, in a study conducted in the United States, the prevalence of depression in breast cancer patients was found to be 44%, which is much lower compared to the depression rate in our study^12^. There are numerous factors that can influence the development of depression in individuals diagnosed with breast cancer: age, marital status, educational level, income level, a family history of breast cancer, stage of the disease, menopause status, body image, and the effects of chemotherapy on fertility and sexual life. The presence of several factors influencing depression among patients can explain the observed variations in depression prevalence across different geographies and studies^13^. Our results showed that there was no relationship between the type of mastectomy procedure and depression. Similarly, a meta-analysis and systematic review reported that whether the mastectomy procedure was radical, partial, or reconstructive did not affect depression^14^. However, another additional study indicated that radical mastectomy was correlated with a higher prevalence of depression than partial mastectomy^15^. Undoubtedly, it is difficult to determine whether the association between depression and the type of mastectomy is related to the surgical method or the cancer itself. In this study, we determined that marital status did not affect depression. However, depression decreased as the age of the participants increased. Additionally, depression was more common in premenopausal patients than in menopausal patients. A negative correlation was detected between depression and the number of children, but this did not reach statistical significance. Our findings concerning age, menopausal status, and the number of children supported the literature^16-18^. Young patients’ concerns about deterioration in their body image, sexual problems, early menopause, and not being able to have children, as well as the potential recurrence of the disease, may be the reasons for the high prevalence of depression in these patients. The emotional support offered by children to their mothers may be important for stress management. Thus, the higher prevalence of depression in those with a small number of children can be explained by the limited support they receive. In our study, there was a negative correlation between income level and depression, but this did not reach the statistical significance level. Upon reviewing the existing literature, it becomes evident that individuals with a lower income are more prone to experiencing depression^19-21^. We also compared depression and sleep quality between breast cancer patients undergoing neoadjuvant and those undergoing adjuvant chemotherapy and detected a higher prevalence of depression in the former. The results of this study showed that 79.7% of the participants had poor sleep quality. Several factors can potentially influence sleep quality, including the sleep quality scale used, the sociocultural and economic levels of patients, the surgical procedure applied, and the stage of the disease^22-24^. In our study, we also found a moderately positive correlation between depression and poor sleep quality. Disturbances in sleep quality affect the patient's daily mood and create a tendency toward depression. Therefore, evaluating sleep disorders and depression together by taking a holistic approach will greatly facilitate the management of these patients. This study has certain limitations. The single-center design and the low educational and income levels of the majority of patients make it difficult to generalize the findings to the whole country. Depression was statistically significantly different between those receiving neoadjuvant and adjuvant treatment, but the small number of patients between these two groups can be considered another limitation.

## CONCLUSION

Depression and poor sleep quality directly affect breast cancer patients’ adherence to treatment, length of hospital stay, and survival. Surgeons, medical oncologists, radiation oncologists, nurses, and psychologists should be alert and cooperate in the management of breast cancer cancers starting from the diagnosis, given the very high rates of depression and sleep disorders in this patient population.
